# Estimation of Tibiofemoral Joint Contact Forces Using Foot Loads during Continuous Passive Motions

**DOI:** 10.3390/s22134947

**Published:** 2022-06-30

**Authors:** Yunlong Yang, Huixuan Huang, Junlong Guo, Fei Yu, Yufeng Yao

**Affiliations:** 1 State Key Laboratory of Robotics and System, Harbin Institute of Technology, Harbin 150001, China; 19b308019@stu.hit.edu.cn; 2 Orthopaedical Rehabilitation Centre, Weihai Municipal Hospital, Weihai 264209, China; huixuanh@yeah.net; 3 Department of Mechanical Engineering, School of Naval Architecture and Ocean Engineering, Harbin Institute of Technology (Weihai), Weihai 264209, China; junlongg@hit.edu.cn; 4 Department of Neurology, The First Affiliated Hospital of Dalian Medical University, Dalian 116011, China; yufei_2016@126.com; 5 Tianzhi Institute of Innovation and Technology, Weihai 264209, China

**Keywords:** tibiofemoral forces, ranges of motion, rehabilitation, CPM

## Abstract

Continuous passive motion (CPM) machines are commonly used after various knee surgeries, but information on tibiofemoral forces (TFFs) during CPM cycles is limited. This study aimed to explore the changing trend of TFFs during CPM cycles under various ranges of motion (ROM) and body weights (BW) by establishing a two-dimensional mathematical model. TFFs were estimated by using joint angles, foot load, and leg–foot weight. Eleven healthy male participants were tested with ROM ranging from 0° to 120°. The values of the peak TFFs during knee flexion were higher than those during knee extension, varying nonlinearly with ROM. BW had a significant main effect on the peak TFFs and tibiofemoral shear forces, while ROM had a limited effect on the peak TFFs. No significant interaction effects were observed between BW and ROM for each peak TFF, whereas a strong linear correlation existed between the peak tibiofemoral compressive forces (TFCFs) and the peak resultant TFFs (R^2^ = 0.971, *p* < 0.01). The proposed method showed promise in serving as an input for optimizing rehabilitation devices.

## 1. Introduction

The continuous passive motion (CPM) device for the lower limb is a motorized device that passively moves the knee joint through a preset range of motion. It is widely used in the rehabilitation process of knee arthroplasty (KA), early rehabilitation of stroke, and other knee surgeries [1,2,3]. The CPM devices are usually used to eliminate swelling, heal joint soft-tissue, restore joint flexibility and function [4], and reduce the workload of physiotherapists [5]. However, its effectiveness and discomfort have been controversial [6,7]; for example, patients experience pain during rehabilitation due to stretching surgical wounds [8].

A conventional CPM device with a linear electrical drive affects the foot [9] and causes impact loading [10] because of the lack of a bionic design. In recent years, pneumatic actuators [11,12] and optimization of the motors’ location [13,14,15] have been applied to improve the comfort and safety of CPM devices to some extent. However, no conclusive consensus has been reached on reducing impact force because the mechanism for these improvements is still not well understood, and systematic studies of tibiofemoral forces (TFFs) during CPM cycles are lacking. The postoperative rehabilitation of fractures and KA are sensitive to knee joint stress [16]; thus, it is necessary to quantify and decrease this stress during early rehabilitation [17,18]. In addition, the estimated TFFs can be used to plan rehabilitation treatments and monitor abnormal forces during the rehabilitation process. Moreover, TFFs can also be used to optimize the design of CPM devices. However, neither the changing trend of the foot pressure nor the effects of ranges of motion (ROM) on TFFs during CPM cycles have been investigated.

Although in vivo and in vitro studies have estimated TFFs, most of these systems are non-portable, non-real-time assessment, and laboratory-based. An implantable telemetry electronic prosthesis was designed to directly measure TFFs during the activities of daily living [19,20]. Moreover, musculoskeletal models [21], finite element models [22], and wearable inertial devices [23,24] have been applied to estimate TFFs. However, without complex experimental settings, it is difficult to use an electromyogram-driven musculoskeletal model, smart implant, or finite element model to analyze the influence of external forces on TFFs.

Pressure-sensitive insoles have been widely used in both gait assessment [25,26] and the measurement of plantar pressure during rehabilitation [27,28,29]. A general two-dimensional knee model [30,31] and pressure-sensitive insoles were employed in this study to estimate TFFs. The aims of this study were as follows: (1) establishing a general two-dimensional mathematical knee model, (2) estimating TFFs using the established model and the directly measured foot pressure, and (3) exploring the influence of ROM and body weight (BW) on the peak resultant TFFs and peak tibiofemoral shear forces (TFSFs).

## 2. Materials and Methods

### 2.1. Participants

The data were collected from eleven healthy male participants (Table 1) with no disorder of the lower extremities or any symptoms of muscle soreness, where L indicated the distance between the plantar and the knee axis. All participants were informed about the test procedures, and they provided informed written consent prior to the study. The study was conducted in accordance with the Declaration of Helsinki, and the protocol was approved by the local Ethics Committee (Ethics Committee of Weihai Municipal Hospital; Ref. No. 2020055). The participants were divided into three groups according to their BWs.

### 2.2. Experimental Data Collection

Because of the limitation of the CPM device, the ROM of the lower extremities ranged from 60° to 120° at intervals of 20°. Each trial was repeated four times and included more than three cycles. The participants were required to relax their bodies and refrain from voluntary movement during CPM cycles, and only the left leg was tested. All the experiments were controlled by a physical therapist (not a member of the authorship), and the foot loads and joint angles were measured simultaneously.

**Figure 1:**
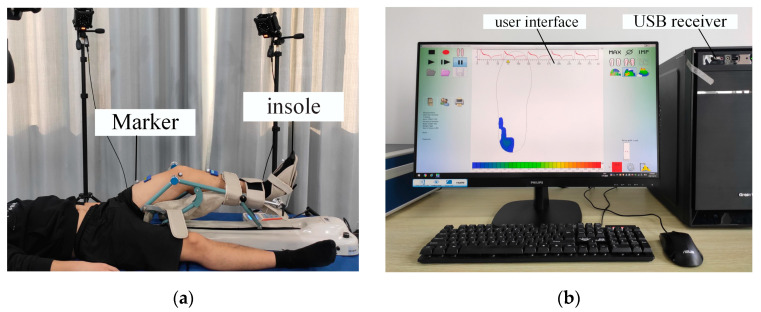
Acquisition system used in the experiments. (**a**) Sketch of the experimental setup; (**b**) graphical user interface and receiving device.

The CPM device (The KH-11, Model JK-A lower-limb CPM; CANWELL Technologies, Jinhua, China) contained a handheld digital display unit. The display angle of the CPM device was verified with an inertial sensor. To avoid fatigue, each experiment was limited to 5 min, and the interval was more than 5 min. A motion-capture system with eight cameras (Mars2H, Nokov, Beijing, China, sampling rate 50 Hz) was used to estimate the knee joint angle (*ψ*_k_) and the hip joint angle (Figure 1). The CPM device moved at a relatively low speed of 6°/s to decrease the influence of the inertia of the CPM on the foot load.

**Figure 2:**
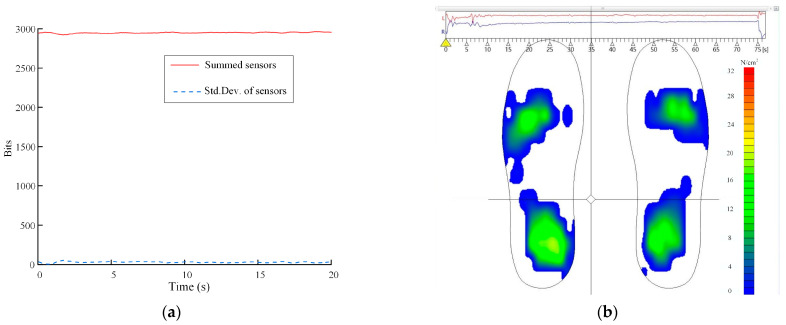
Curve of pressure soles during calibration. (**a**) Curve of the sensors summed for participant A4; (**b**) Example curve of foot pressure.

The pressure between the foot and the support plate was measured using a plantar pressure system (T&T Medilogic Medizintechnik GmbH, Schönefeld, Germany, 50 Hz) [32]. The plantar pressure insole started to record the pressure data before starting the CPM machine. The insole was selected according to the foot length (sizes of 39–44 were available) and calibrated using participants’ weights [25,29,33] before the experiment. Participants’ weights were used to calibrate the digital output of sensors while the participant was standing still. Each participant stood still for more than 60 s, and the digital output was subtracted from the time series. Each trial was repeated three times. The calibration factor (*q_x_*) can be estimated using Equation (1):

(1) $$q_{x}=[\sum_{i=1}^{n_{i}}\left(l_{i,j}\frac{64\;}{255}\right)1.125]\;/W_{x}g$$

where *W_x_* is the mass of participant *x*, *g* is the acceleration of gravity, and *l_i,j_* denotes the 0–255 digital value output of sensor *i* at instant *j*. The variability of the active sensors summed for participant A4 is shown in Figure 2a, which shows that the variability is small.

### 2.3. Biomechanical Model

Four assumptions were made to consider the anatomical structure and physiological characteristics of the knee joint to establish a general lower limb model.

**Figure 3:**
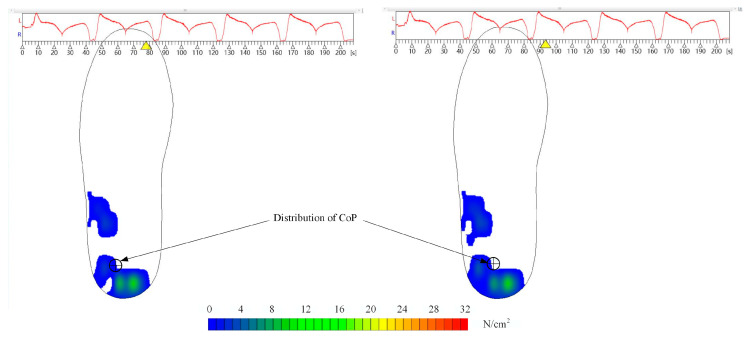
CoP distribution of the foot pressure.

**Figure 4:**
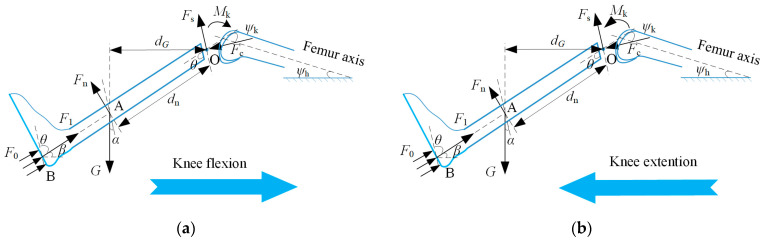
Free-body diagram of the femur and tibia during CPM cycles. (**a**) *F*_0_, the force generated by plantar pressure; *F*_1_, the resultant force of *F*_0_; *F*_n_, the normal force imparted by the tibia brace; *G*, the weight of the leg–foot system; *M*_k_, the passive torque of the knee generated by the tissue; *β*, the angle of tibia long-axis incline; *α*, the angle between *F_s_* and *G*; *d*_n_, the distance from the center of mass of the leg–foot system to the knee axis [36]; *d_G_*, the moment arm of *G* around knee axis; *ψ*_k_, the knee flexion angle; *F_c_*, the tibiofemoral compressive force; *F_s_*, the tibiofemoral shear force; *ψ*_h_, the hip flexion angle. (**b**) Forces and distances for the free-body diagram of the femur and tibia during knee extension.

(1) The lower limb was regarded as a rigid body model in the sagittal plane, the lower leg and foot (foot–leg system) were regarded as one body, and the average angle (θ) between the tibial plane and its long axis was 76.8° [30].
(2) The center of pressure (CoP) distribution of insole measurements in the insole coordinate system was around the tibial axis, with the ankle angle kept at 0° (Figure 3). The moments induced by the position error of CoP could be presented by *M_e_* in Nm.
(3) The center point, O, of the intercondylar contact coincided with the origin of moments and force, guaranteeing that the moment generated by the pressure and shear stresses was equivalent to zero [31,34].
(4) The knee joint had no active force and was affected only by the passive torque, *M*_k_, generated by the tissue [35]. It moved in the sagittal plane with only one degree of freedom, and the knee axis was unicentric (Figure 4).

Considering the low speed of the rehabilitation movement, the lower limb was regarded as quasi-static, and point O was selected as the origin of the torque calculation. The following equations provide the resultant force and moment around O:

(2) $$\left\{ \begin{array}{l} F_{n}d_{n}+M_{k}+M_{e}=Gd_{G}\;(\mathrm{knee}\;\mathrm{flexion})\\ F_{n}d_{n}-M_{k}+M_{e}=Gd_{G}\;(\mathrm{knee}\;\mathrm{extention}) \end{array}\right.$$

where *d*_n_ denotes the distance from the center of mass of the leg–foot system to the knee axis; for more details, please refer to [36]. *G* is the gravity of the foot–leg system, which was estimated using kinematic and anthropometric data [37]. *d_G_* could be calculated using Equation (3):

(3) $$d_{G}=d_{n}\cos\beta$$

The passive torque *M*_k_ (Nm) of the knee could be calculated using:

(4) $$M_{k}=e^{1.8-0.0352\psi_{k}+0.0217\psi_{h}}-e^{-3.971+0.0495\psi_{k}-0.0128\psi_{h}}+e^{2.22-0.15\psi_{k}}-4.82\;$$

where *ψ*_k_ and *ψ*_h_ are knee and hip joint angles, respectively, measured by the motion-capture system [35]. Angles are presented in degrees. *F*_1_ in N was the resultant measured plantar force at an instant and can be calculated using Equation (5):

(5) $$F_{1}=\left(\sum_{i=1}^{n_{i}}\left(l_{i,j}\frac{{64\;N/\mathrm{cm}}^{2}}{255}\right)1{.125\mathrm{cm}}^{2}\right)/q_{x}$$

where *l_i,j_* denotes the 0–255 digital value output of sensor *i* at instant *j* [32], and *q_x_* is the calibration factor.

*M_e_* can be calculated using Equation (6):

(6) $$M_{e}=F_{1}y$$

where *y* denotes the CoP location shift in the sagittal plane. The tibiofemoral compressive force *F_c_* and the tibiofemoral shear force *F_s_* could be calculated using Equation (7).

(7) $$\left\{ \begin{array}{l} F_{s}=F_{1}\cos\theta +F_{n}\sin\theta -G\cos\alpha \\ F_{c}=F_{1}\sin\theta +F_{n}\cos\theta +G\sin\alpha \end{array}\right.\;(\alpha =\left\{ \begin{array}{l} \frac{\pi }{2}-\theta +\beta ,\beta >0\\ \theta -\frac{\pi }{2}+\beta ,\beta <0 \end{array}\right.)$$

All equations were combined and numerically solved using the computational software Maple (Maple 2020).

### 2.4. Data Analysis

The duration of each CPM cycle was normalized to 100% for comparison among conditions and across participants. All statistical analyses were performed with SPSS 24.0 (version IBM SPSS 24; SPSS Inc., Armonk, NY, USA). A one-way analysis of variance (ANOVA) was used to test the significance of the differences in forces among the various ROM, and Bonferroni post hoc tests were performed if the ANOVA result was significant (*p* < 0.05). The effects of BW and ROM on the peak resultant TFFs and peak TFSFs were analyzed by two-way ANOVA. Significant interactions and main effects were post hoc analyzed using Bonferroni correction.

## 3. Results

### 3.1. Typical Peak Resultant TFFs

**Figure 5:**
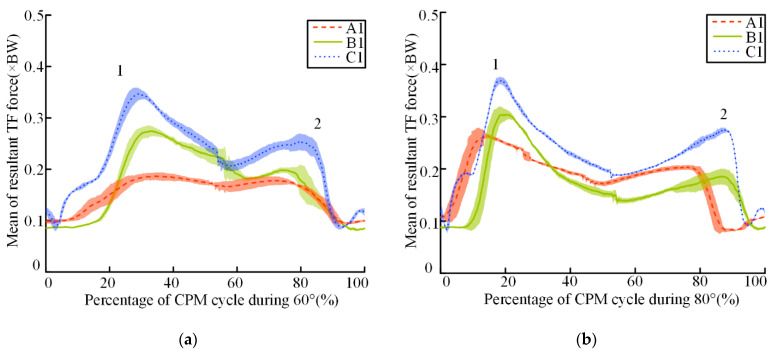

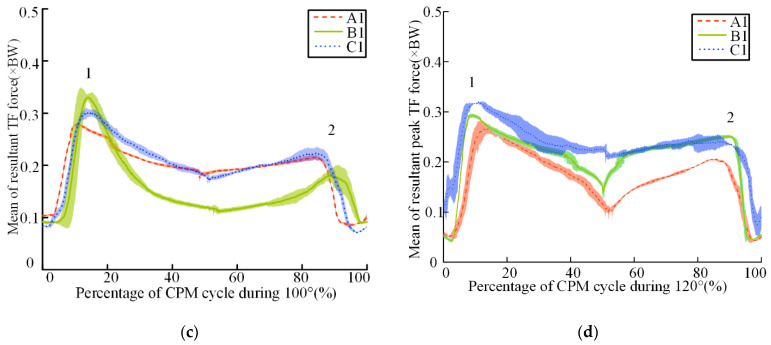
Mean (and standard error) of the estimated resultant TFFs from three subjects during typical CPM cycles. (**a**–**d**) represent 60°−120°, respectively; 1 = first major peak during knee flexion; 2 = second major peak during knee extension. The shaded area represents ±1 standard deviation.

Representative CPM cycles from three participants (that is, A1, B1, and C1) demonstrated the existence of two major peak forces during each complete cycle (Figure 5), with forces represented as the multiples of BW. Although the peak resultant TFFs of these participants were different, the changing trend of the estimated TFFs was similar. Figure 5 depicts the major peak knee contact forces during a CPM cycle ranging from 0.24 × BW during cycles of 60° to 0.43 × BW at a ROM of 120°. The TFFs during a cycle were asymmetrical, and the values of the peak resultant TFFs during knee flexion were higher than those during knee extension. In all cases, the first peak force appeared when the knee flexion approximated 20 degrees.

**Figure 6:**
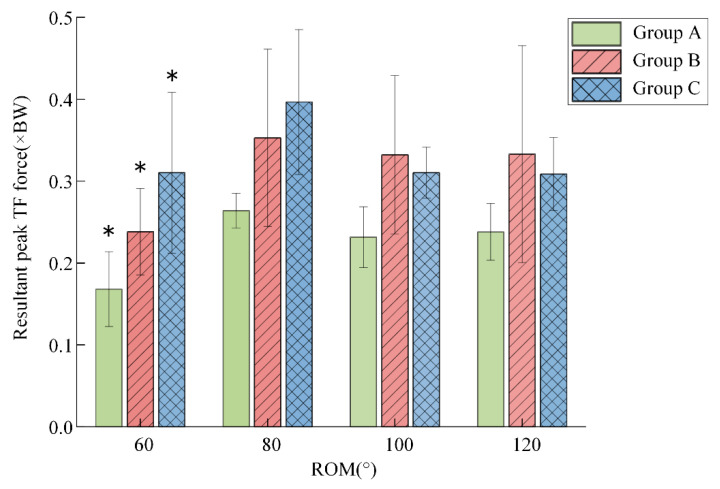
Mean of the major peak resultant TFFs for four ROM across CPM cycles. All significant results were compared with the ROM of 80° (* *p* < 0.05).

The intragroup statistical analysis of the peak resultant TFFs of the three groups was conducted to evaluate the effect of ROM alone (Figure 6). Significant differences were observed between the ROM of 60° and that of 80° (*p* < 0.05) for all groups. For each group, one-way ANOVA intragroup comparison indicated that the lowest mean value of the peak resultant TFFs was recorded during the ROM of 60°, varying from 0.16 (BW) to 0.31 (BW). Moreover, the results showed that the highest mean peak resultant TFFs was observed during the ROM of 80° rather than during the ROM of 120°. When the ROM was larger than 80°, the mean peak resultant TFFs experienced a slight decrease.

### 3.2. TFCFs and TFSFs

**Figure 7:**
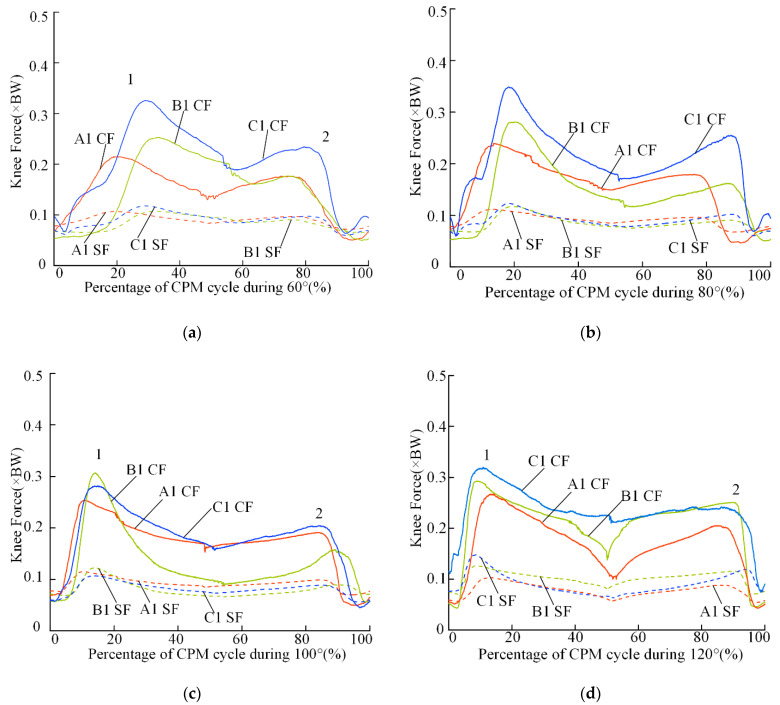
Mean of the estimated TFCFs (solid lines) and TFSFs (dashed lines) from participants during three repeated typical CPM cycles at different ROMs; 1 = First major peak during knee flexion; 2 = second major peak during knee extension. A1 CF represents the compressive forces of A1, and A1 SF represents the shear forces of A1. (**a**–**d**) represent 60°–120°, respectively.

Figure 7 presents the mean estimated values of the TFCFs and TFSFs in three participants (that is, A1, B1, and C1); significant differences were observed between the peaks of the TFCFs and the TFSFs (*p* < 0.05). The average value of the peak compressive forces was approximately two or three times the magnitude of the peak shear forces. Compared with the TFSFs, the TFCFs changed significantly. Similar to the trend of the resultant TFFs, the peaks of the TFSFs during knee flexion were typically higher than those during knee extension.

### 3.3. Interactions of BW and ROM on the Peak Resultant TFFs and TFSFs

The two-way ANOVA indicated no significant interaction effects between BW and ROM for the peak resultant TFFs. Generally, the mean value of the peak resultant TFFs during the CPM motion arc of 60° was the lowest (Table 2) and was significantly different from that during the CPM motion arc of 80° (*p* < 0.05). Moreover, the BW had significant main effects on the major peak of the resultant TFFs (*p* < 0.01). The peak resultant TFFs increased with the BW, which caused a significant difference between groups A and B/C (*p* < 0.01).

The peak resultant TFFs were in a significant linear relationship with the peak TFCFs (R^2^ = 0.971, *p* < 0.01), which was obtained using linear regression analysis, indicating the approximate statistical properties of the TFFs and TFCFs. Additionally, no significant interactions were detected between BW and ROM for the peak TFSFs (two-way measure ANOVA), whereas the BW had a significant main effect on the peak TFSFs when comparing group A with B/C (*p* < 0.05). Compared with the mean value of the peak resultant TFFs, the mean peak of TFSFs was almost one-third to one-half of the former (Table 2). Details of the data for plantar pressure and video of data acquisition experiments are reported in the Supplementary Material.

## 4. Discussion

This exploratory study examined the effects of BW and ROM on the changing trend of peak resultant TFFs and peak TFSFs during CPM cycles. The ROM had a limited effect on the peak resultant TFFs, while the BW had a significant main effect on the peak resultant TFFs and peak TFSFs. No significant interactions between BW and ROM were witnessed, but a strong linear correlation between the peak resultant TFFs and peak TFCFs was observed.

The results in Figure 6 indicate that TFFs did not continually increase with ROM; the biggest mean peak resultant TFFs occurred during the ROM of 80° rather than the ROM of 120°. The reason was not fully understood, but the mechanisms of the therapeutic action of CPM and physiologic explanations might account for it. Few studies have systematically investigated the effect of ROM on TFFs during CPM cycles. We cannot directly compare our results with the findings of other studies carried out without rehabilitation devices, such as walking and running. However, previous studies seem consistent with our findings to some extent. Bergmann et al. [38] showed that the peak resultant force occurred with a flexion angle of 94° rather than a bigger knee-flexion angle. In addition, the peak resultant TFFs during the flexion phase were smaller than those during the extension phase, indicating that the resultant TFFs in a CPM cycle were asymmetric, which was in agreement with a prior study of the intra-articular pressure of the human knee during CPM [39]. It is worth noting that the observed trend may be affected by the motion trajectory of rehabilitation devices; thus, our method can serve to plan the trajectory of rehabilitation robots.

Unlike the peak TFCFs, the peak TFSFs were not affected by ROM. The peak TFSFs were substantially lower than the peak TFCFs; a similar pattern of results was observed during walking [40] and isokinetic knee extension [31]. However, the peak TFSFs in the present study had relatively low levels compared with previously reported values (5% vs. 25%). This could be explained since the knee extensor muscles and patellar tendon of participants in our study were not activated during the knee joint passive motion. Thus, although the location of the tibiofemoral contact point (C) and the knee axis were not exactly coincident as hypothesized, the error induced by this bias would be lower than 5%.

The BW was the main factor affecting the peak resultant TFFs, and the peak resultant TFFs increased with the BW when comparing group A with groups B and C. The TFFs increased with the BW of leg–foot systems. This changing trend was consistent with the results obtained in previous studies [19,41] in which the knee joint contact forces depended on BW. However, the predicted peak TFFs (Table 2) in this study were considerably lower than the reported results by Damm et al. [19] (TFFs ≥ 2.5 BW). Compared with the study [19], the load only exerted by the leg may account for the difference in TFFs. Obesity increased the prevalence of diagnosed knee abnormalities [42] and might impair the early outcome of KA [43,44]. Thus, weight management before KA might decrease the pain during CPM after KA, which coincided with the results reported in previous studies [45,46]. In addition, under various BWs, the changing trends of the estimated forces versus time in CPM cycles were similar, and this was similar to that observed during walking [47,48].

**Figure 8:**
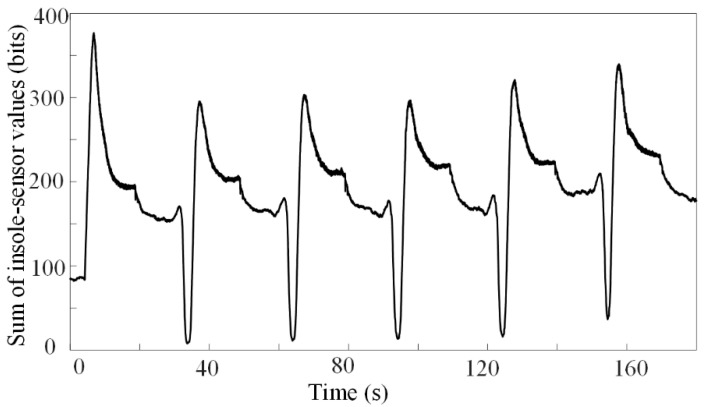
Measured plantar pressure curves.

The directly measured plantar pressure changed with time, as shown in Figure 8, indicating that the soft tissues might deform along the tibial axis; otherwise, the curve should change symmetrically and smoothly during each CPM cycle. The CPM equipment could not align with complex knee movements [49,50] and stretch surgical wounds [8]. Hence, patients suffered from pain during CPM cycles [51]. Therefore, reducing the deformations of soft tissues by improving kinematic compatibilities might decrease pain during CPM cycles. The estimated TFFs can be utilized as optimization metrics to minimize the risk of a knee injury. In conclusion, reducing the effect of CPM on surgical wounds during knee rehabilitation is one key aspect of our future investigations. Additionally, exploring the skin deformation of patients and detecting the relationship between skin deformation and pain during CPM cycles will be the focus of future studies.

Overall, the proposed mathematical method may decrease the performance of some movements but provides the advantage that not every movement must be modeled. This generic model is easier to apply in clinical and home-based rehabilitation owing to the ease of measurement of BW and height compared with the musculoskeletal model [47] and the visual motion capture system [29,52]. Measured foot pressure has been used to evaluate abnormal walking behavior [53,54] and the CoP of active motions [28]. Furthermore, the estimated TFFs using measured foot pressure can act as a biofeedback method to monitor abnormal force during rehabilitation exercises to prevent injuries, implying that the proposed method could be applied in both active and passive motions.

This study has a few limitations. First, most CPM machines are similar in mechanical design. However, only one brand of CPM machine was tested. Second, considering that the CPM has a wide range of users, we recruited only healthy male individuals to better explore the changing trend of TFFs. To compensate for this difference between healthy male individuals and KA population [55], this method can also be applied to patients after KA by modifying the values of *θ* and *M*_k_. In the future, both healthy individuals and patients undergoing knee surgeries in different stages will be recruited to verify the proposed method. Third, the numbers cited for the mass, location of the center of mass, and passive elastic joint moments were estimated statistically from sampled individuals. Thus, they were regarded as estimates.

## 5. Conclusions

This study described a novel integrated approach with a general two-dimensional knee model and pressure-sensitive insoles to explore the changing trend of TFFs versus ROM and BW during CPM cycles. The ROM had a limited effect on the peak resultant TFFs, whereas TFFs did not increase linearly with ROM. The BW had a significant positive effect on both TFSFs and TFFs. No significant interactions between BW and ROM were observed. The proposed method may serve as an input for optimizing rehabilitation devices. Moreover, this method showed promise for clinical and long-term home-based rehabilitation owing to its portable and configurable properties.

## Figures and Tables

**Table 1:** Overview of participant data.

Group		L (mm)	Weight (kg)	Age (Years)	Height (cm)
A (60−68 kg)	A1	485	61.9	22	178
	A2	464	63.8	26	170
	A3	465	63.9	26	171
	A4	478	64.8	25	175
B (70−78 kg)	B1	480	70.7	26	176
	B2	510	73.4	26	185
	B3	490	75.2	23	180
C (80−88 kg)	C1	465	82.7	23	172
	C2	485	83.3	27	178
	C3	481	86.3	23	176
	C4	465	87.8	26	170

**Table 2:** Mean values (+SD) of maximum resultant TFFs, TFCFs, and TFSFs (×BW) during various testing conditions.

Max Force (×BW)	Group	60° (SD)	80° (SD)	100° (SD)	120° (SD)
Resultant TF force	A	0.16 (0.05)	0.26 (0.02)	0.23 (0.04)	0.24 (0.03)
	B	0.24 (0.05)	0.35 (0.11)	0.33 (0.09)	0.33 (0.13)
	C	0.31 (0.10)	0.40 (0.09)	0.31 (0.03)	0.31 (0.04)
Compressive force	A	0.16 (0.04)	0.26 (0.04)	0.21 (0.04)	0.21 (0.04)
	B	0.22 (0.05)	0.34 (0.08)	0.30 (0.09)	0.31 (0.12)
	C	0.30 (0.11)	0.36 (0.10)	0.29 (0.03)	0.29 (0.05)
Shear force	A	0.08 (0.03)	0.09 (0.03)	0.10 (0.04)	0.10 (0.03)
	B	0.10 (0.01)	0.12 (0.02)	0.12 (0.03)	0.11 (0.05)
	C	0.12 (0.01)	0.13 (0.02)	0.11 (0.01)	0.11 (0.01)

## Supplementary Materials

The following supporting information can be downloaded at: https://figshare.com/articles/dataset/Supplementary_Materials/19766644/2 (accessed on 15 May 2022). Table S1. Data for Figure 4 and Figure 6. (XLSX); Table S2. Data for plantar pressure of A1, B1, and C1. (CSV); Video S1. Video of data acquisition experiments (MP4).

## Abbreviations

CPM	continuous passive motion
KA	knee arthroplasty
TFFs	tibiofemoral forces
TFCFs	tibiofemoral compressive forces
TFSFs	tibiofemoral shear forces
BW	body weight
CoP	center of pressure
*M*_k_	passive torque generated by the tissue
ANOVA	analysis of variance
